# Altered anti-viral immune responses in monocytes in overweight heavy drinkers

**DOI:** 10.1016/j.isci.2023.107133

**Published:** 2023-06-15

**Authors:** Adam Kim, Martí Ortega-Ribera, Megan R. McMullen, Annette Bellar, Moyinoluwa Taiwo, Vai Pathak, David Streem, Jaividhya Dasarathy, Nicole Welch, Srinivasan Dasarathy, Vidula Vachharajani, Laura E. Nagy

**Affiliations:** 1 Northern Ohio Alcohol Center, Department of Inflammation and Immunity, Cleveland Clinic, Cleveland, OH, USA; 2 Department of Medicine, University of Connecticut Health Center, Farmington, CT, USA; 3 Lutheran Hospital, Cleveland Clinic, Cleveland, OH, USA; 4 Department of Family Medicine, MetroHealth, Cleveland, OH, USA; 5 Department of Gastroenterology and Hepatology, Cleveland Clinic, Cleveland, OH, USA; 6 Department of Molecular Medicine, Case Western Reserve University, Cleveland, OH, USA; 7 Department of Critical Care Medicine Cleveland Clinic Respiratory Institute, Cleveland, OH, USA

**Keywords:** Molecular biology, Immunology, Omics, Transcriptomics

## Abstract

Alcohol abuse causes increased susceptibility to respiratory syndromes like bacterial pneumonia and viral infections like SARS-CoV-2. Heavy drinkers (HD) are at higher risk of severe COVID-19 if they are also overweight, yet the molecular mechanisms are unexplored. Single-cell RNA-sequencing (scRNA-seq) was performed on peripheral blood mononuclear cells from lean or overweight HD and healthy controls (HC) after challenge with a dsRNA homopolymer (PolyI:C) to mimic a viral infection and/or with lipopolysaccharide (LPS). All monocyte populations responded to both PolyI:C and LPS with pro-inflammatory gene expression. However, the expression of interferon-stimulated genes, essential for inhibiting viral pathogenesis, was greatly reduced in overweight patients. Interestingly, the number of upregulated genes in response to the PolyI:C challenge was far greater in monocytes from HD compared to HC, including much stronger pro-inflammatory cytokine and interferon-γ signaling responses. These results suggest that increased body weight reduced anti-viral responses while heavy drinking increased pro-inflammatory cytokines.

## Highlights

• Single-cell RNA-seq of PBMCs isolated from heavy drinkers and overweight patients
• Overweight patient monocytes had reduced interferon-stimulated gene expression
• Heavy drinker monocytes were hyper pro-inflammatory after PolyIC and LPS
• IFNG signaling was much higher in heavy drinkers

## Graphical abstract

**Figure:**
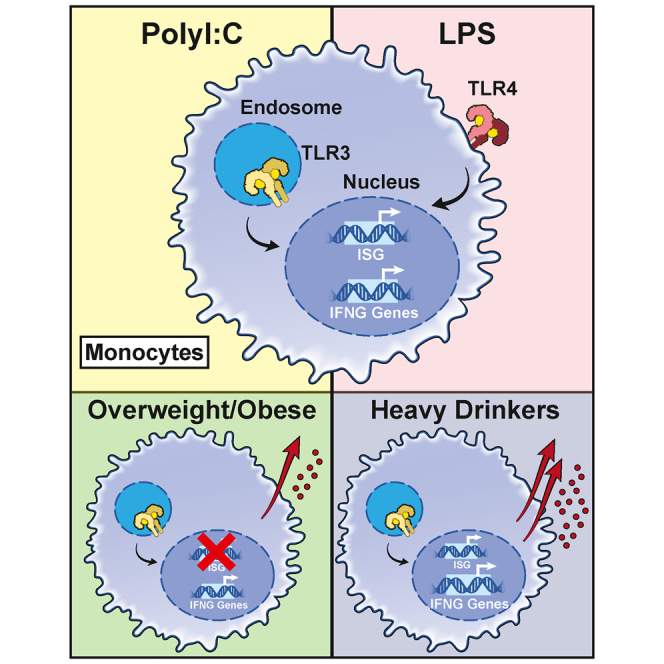

## Introduction

Chronic alcohol consumption and heavy drinking increases the severity and worsens outcomes of a number of infectious diseases, including bacterial pneumonia and tuberculosis, and many viral diseases, such as hepatitis B, hepatitis C, and HIV.^1^^,^^2^ Alcohol use disorder, reported by 1/8^th^ critically ill patients, is an independent risk factor for death from sepsis.^3^ The SARS-CoV-2 virus, which caused the worldwide COVID-19 pandemic, causes severe disease among patients who are overweight or obese.^4^^,^^5^ Recent studies have also found that heavy drinkers (HD), especially those who are also overweight or obese, are prone to increased risk for severe COVID-19 and death.^6^^,^^7^ Together, these studies implicate immune dysfunction caused by obesity and alcohol misuse to be significant contributors to viral disease severity.

Viral infections, such as COVID-19, lead to a robust antiviral response, specifically through type I and type III interferons.^8^ This interferon response triggers upregulation of interferon-stimulated genes (ISGs) whose primary role is to inhibit viral entry and replication in cells.^9^^,^^10^ As part of the general immune response, interferons also upregulate a number of cytokines and chemokines, which activate and recruit other immune cells, thus amplifying the signal.^11^ All of these immune responses (interferons, cytokines, and chemokines) are also activated during bacterial infections via lipopolysaccharide (LPS) through toll-like receptor 4 (TLR4) signaling.^12^^,^^13^ Viral infections, such as influenza, can be further complicated by bacterial infections leading to pneumonia, while COVID-19 appears to have fewer bacterial co-infections compared to influenza.^14^ However, patients with COVID-19 and other comorbidities such as obesity are highly prone to bacterial infections significantly worsening outcomes.^15^ How viral immune responses intersect with bacterial immune responses is complex and likely dependent on many factors including type of virus and bacteria, site of tissue damage, and strength of the immune response.

SARS-CoV-2 is a virus that can evade a strong interferon response as compared to other viruses like influenza.^16^^,^^17^ Patients with severe COVID-19 infections have altered type-I, -II, and -III interferon responses in both plasma and in peripheral blood mononuclear cells (PBMCs).^18^^,^^19^^,^^20^^,^^21^ The impact of severe COVID-19 on interferon responses is still not well understood. In this context, some studies find decreased type-I interferon responses^18^^,^^22^ while others observe increased expression.^20^^,^^21^ In contrast, studies consistently find increased proinflammatory cytokines such as tumor necrosis factor (TNF), IL-6, and chemokines in severe COVID-19 patients.^18^^,^^20^^,^^21^^,^^22^ Evidence suggests that interferon-γ (IFNG), the type-II interferon, is capable of enhancing viral clearance.^23^^,^^24^^,^^25^ Higher IFNG is associated with lower incidences of lung fibrosis post-COVID, thus making it a possible anti-viral therapeutic.^25^ However, IFNG is actually associated with worsened outcomes in COVID-19 by contributing to inflammation and cytokine storm,^26^^,^^27^ possibly because IFNG can increase myeloid cell activation during toll-like receptor (TLR) signaling.^13^ Thus, IFNG is very likely to be both necessary for viral clearance, but also highly pro-inflammatory, so inhibitors of IFNG would only be a useful therapeutic after infection is resolved. Together, these data suggest that severe COVID-19 is associated with dysfunctional interferon responses, combined with increased pro-inflammatory cytokine responses, leading to uncontrolled viral replication and severe systemic inflammation.

In this report, PBMCs were challenged with PolyI:C to simulate a strong viral infection (via TLR3) and LPS to simulate a strong bacterial infection (via TLR4) to better understand the impact of being overweight and heavy drinking on interferon and cytokine responses. PBMCs were isolated from healthy lean and overweight patients and lean and overweight HD. Using single-cell RNA-sequencing (scRNA-seq), we explore how body weight and heavy drinking impact how diverse monocyte populations respond to these immune challenges and thus make individuals more susceptible to viral and bacterial infections.

## Results

### Proteomics of plasma from severe COVID-19 patients reveal dysregulated IFN responses in obese patients

**Figure 1:**
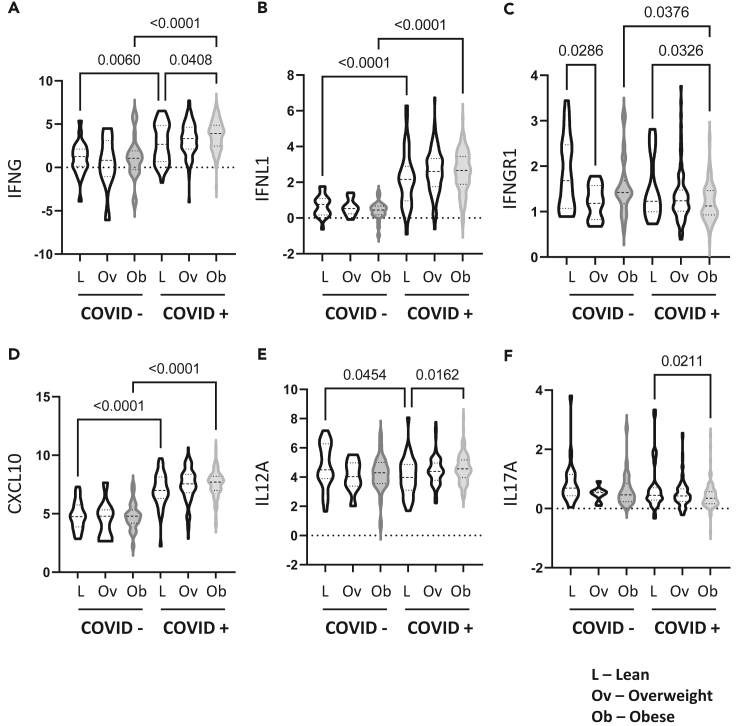
Analysis of the plasma proteome from COVID-19 patients focusing on expression of antiviral-related molecules when comparing lean (L), overweight (Ov), and obese (Ob) patients (A) IFNG, (B) IFNL1, (C) IFNGR1, (D) CXCL10, (E) IL12A, (F) IL17A. Data are normalized protein expression values reanalyzed from Filbin et al.^28^ P = values are indicated above the bars.

Both obesity and heavy drinking worsen outcomes in COVID-19 patients,^4^ so we initially wanted to better understand differences in inflammatory responses between these populations during severe COVID-19 infection. While few clinical studies have assessed drinking status using standard measures, such as AUDIT scores, during COVID-19 infection, body mass index (BMI) data are often recorded, allowing us to stratify data based on body weight. Filbin et al. recently published proteomics data of plasma from hospitalized patients with acute respiratory distress that were then determined to have COVID-19 infection.^28^ Using this dataset, we stratified the data in order to compare lean (BMI <25, n = 23 COVID-, n = 41 COVID+), overweight (BMI 25–30, n = 9 COVID-, n = 96 COVID+), or obese (BMI>30, n = 26 COVID-, n = 123 COVID+) patients.^28^ IFNG and IFNL1 (interferon lambda 1) concentrations were increased in plasma of all COVID+ patients and were further elevated in obese patients, with a modest increase in the overweight group (Figures 1A and 1B). IFNGR1, the receptor for IFNG, was significantly reduced in the obese patients with COVID-19 (Figure 1C). In contrast, CXCL10 was elevated in all COVID+ patients, but was not altered by being overweight or obesity (Figure 1D). Moreover, cytokines IL12A and IL17A were unchanged in the COVID+ samples except for obese COVID+ patients, where IL12A was elevated and IL17A was reduced (Figures 1E and 1F). Together, these data suggest that during severe COVID-19 infection, some, but not all, circulating interferon and pro-inflammatory cytokine concentrations are increased in obese patients, suggesting that worsened outcomes for obese patients are not solely due to exacerbated inflammation.

### scRNA-seq of PBMCs challenged with PolyI:C and LPS

**Figure 2:**
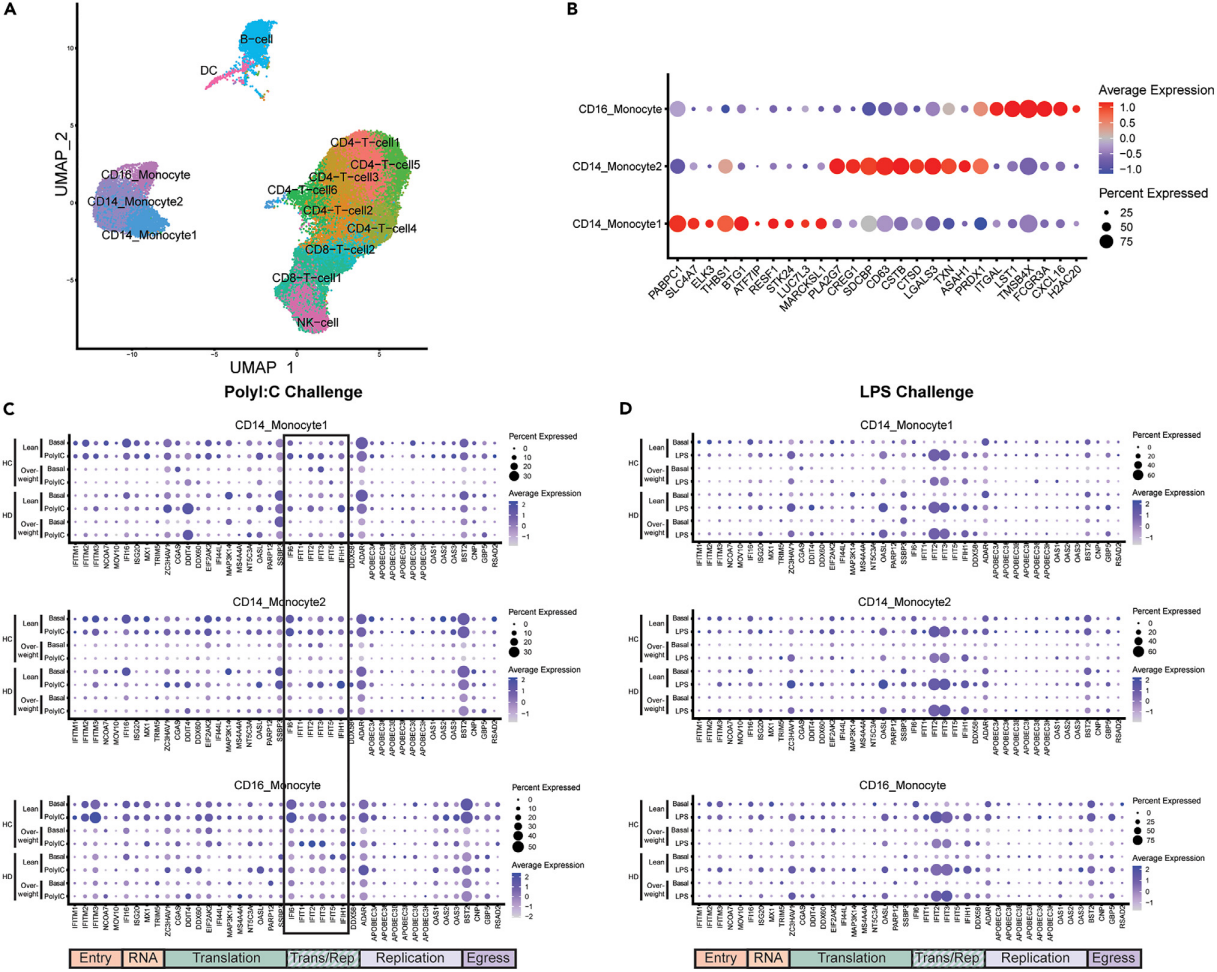
scRNA-seq of PBMCs from HD and HC, lean and overweight patients (A) UMAP plot of all scRNA-seq data showing all PBMC cell types analyzed, including CD4 and CD8 T cells, B cells, NK cells, monocytes, and DCs. (B) Dot Plot showing relative expression and percentage of cells expressing genes specific to each monocyte cluster. (C and D) Expression of ISGs was decreased in monocytes from overweight patients at both baseline and in response to C) PolyI:C but not in response to D) LPS. Dot Plot showing relative expression and percentage of cells expressing each gene. ISGs have been organized based on their role in viral inhibition, including inhibition of viral entry, RNA, translation, replication, and egress. Three monocyte clusters, CD14 monocyte 1, CD14 monocyte 2, and CD16 monocyte, are shown, split based on C) PolyI:C (1.5 μg/mL, 2 h) or D) LPS (10 ng/mL, 1 h) treatment. Black box highlights the IFIT genes.

PBMCs were isolated from female heavy drinkers (HD, AUDIT score >16) and healthy controls (HC), then stratified into lean (BMI<25) and overweight (BMI>25) for each group (Tables S1 and S2, n = 3 for all four groups). PBMCs were challenged *ex vivo* with and without PolyI:C (1.5ug/mL) for 2 h and with and without LPS (10 ng/mL) for 1 h, resulting in 4 comparative conditions: Basal, PolyI:C, LPS, and PolyI:C-LPS (Figure S1A). In this design, we studied individual gene expression responses to PolyI:C or LPS, and the effect PolyI:C pretreatment had on LPS responses. In total, all samples combined, 41,178 cells were sequenced by scRNA-seq and all major cell types expected in PBMCs were recovered (Figure 2A and Table S3). For each patient at baseline, there were no significant differences in the distribution of specific cell types due to being overweight or heavy drinking (Figures S1B and S1C). For this study, we focused on monocytes, identifying 3 distinct clusters which expressed specific marker genes: two CD14 monocyte clusters and one CD16 monocyte cluster (Figure 2B). Differential expression analyses at baseline revealed genes up and downregulated in CD14 monocytes from HD compared to HC, with little overlap between clusters 1 and 2 (Table S4). TREML4 was upregulated in HD in both CD14 clusters and expression on monocytes has been associated with inflammation and increased risk for atherosclerosis.^29^ In CD14 monocyte cluster 1, MARCO, which is often associated with noninflammatory myeloid cells, was downregulated in HD.^30^

### Interferon stimulated genes were reduced in myeloid cells from overweight patients

To better understand immune dysfunction and poor outcomes during viral infections in overweight HD, we interrogated the expression of viral response genes after PolyI:C and LPS challenge. We hypothesized that myeloid cells, being the predominant TLR3 and TLR4 expressing cell types, would have the most robust interferon and cytokine responses to these challenges, especially as these cells were only challenged for a short period of time. From the literature, we gathered a comprehensive list of ISGs that play diverse roles in anti-viral mechanisms, including inhibition of viral entry, mRNA synthesis, translation, replication, and packaging.^9^^,^^10^ In general, ISGs were expressed at far lower levels in monocytes from overweight patients, at baseline and in response to PolyI:C, both in terms of average gene expression and percentage of cells expressing (Figure 2C). HD did not have as many differences regarding the expression of ISGs, but HD who were overweight had similarly low expression of ISGs.

In general, the most upregulated ISGs in response to PolyI:C were the IFIT genes involved in the repression of both viral translation and replication. IFIT genes were especially upregulated in HD (Figure 2C, black box). ISG expression patterns were different between the three monocyte clusters, in both HC and overweight patients. In general, CD14 monocyte 1 expressed the fewest ISGs, even in HC, and in response to PolyI:C; only IFIT2 and IFIT3 were upregulated (Figure 2C). In contrast, CD14 monocyte 2 expressed many ISGs at baseline and did not upregulate them in response to PolyI:C. CD16 monocytes also expressed many ISGs, but in overweight patients, CD16 monocytes upregulated IFIT1, 2, and 3. These were the only ISGs upregulated in any monocyte cluster from overweight patients (Figure 2C).

Even though overall ISG expression was decreased in monocytes from overweight patients, all monocytes robustly expressed pro-inflammatory cytokines including TNF and IL1B, and chemokines including CCL3 and CXCL2, after PolyI:C challenge, suggesting these cells are capable of inducing pro-inflammatory gene expression, but not anti-viral genes (Figure S1D). Interestingly, in response to LPS, monocytes robustly upregulated a specific subset of ISGs (OASL, IFIT2, IFIT3, IFIH1, and ZC3HAV1 [ZAP]) regardless of weight or heavy drinking, and at a much higher level compared to PolyI:C treated cells (Figure 2D). Together, these data suggest that PolyI:C-TLR3 signaling to interferon and ISG expression in all monocyte clusters are negatively affected by weight.

### A small subset of monocytes is highly anti-viral

**Figure 3:**
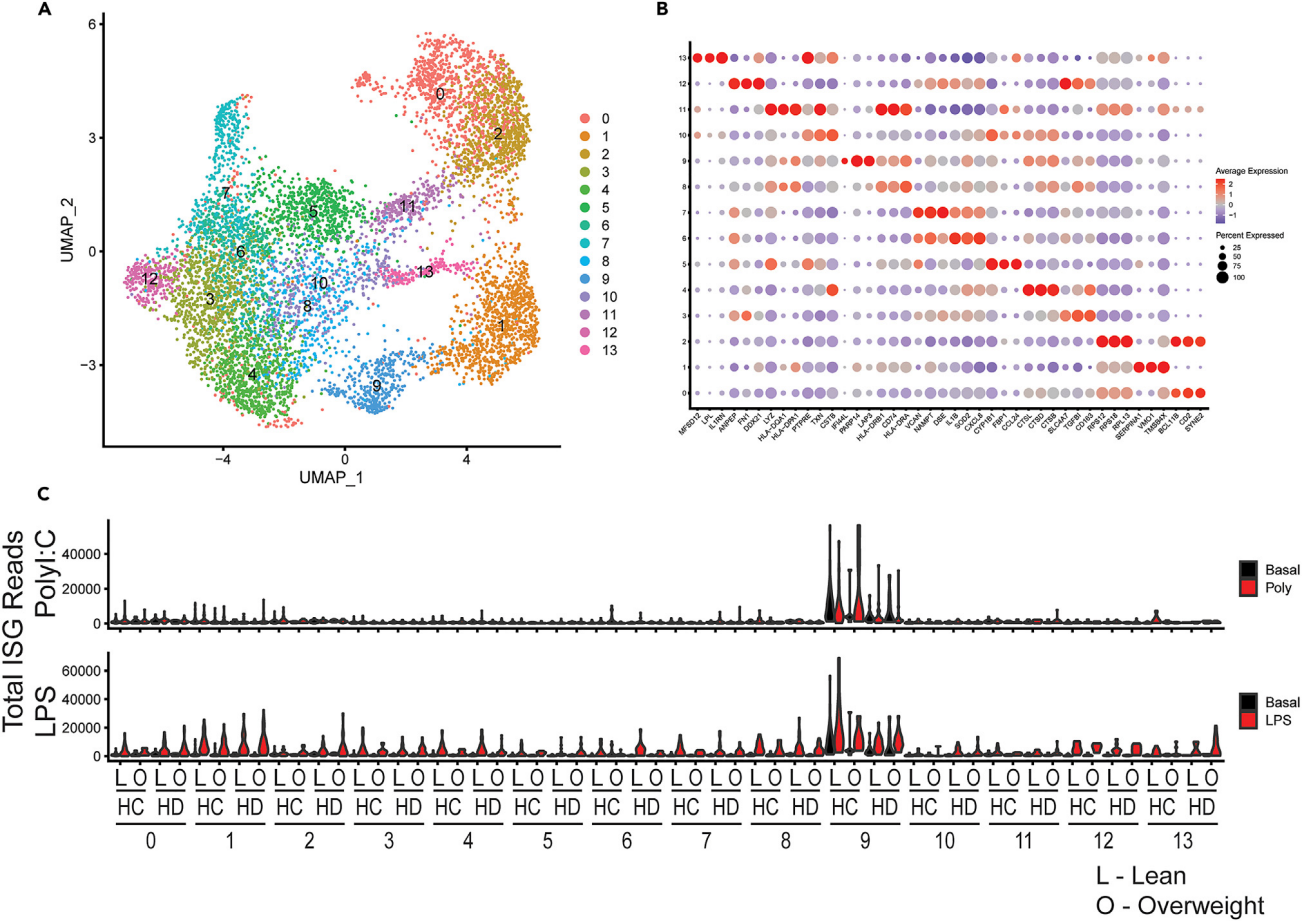
Monocytes have extensive diversity, with a specific cluster (cluster 9) expressing a significant number of anti-viral genes All monocytes (CD14 monocyte 1, CD14 monocyte 2, and CD16 monocyte) were reclustered in order to reveal diversity within the monocyte subpopulation. (A) UMAP showing the 14 different subclusters of monocytes. (B) Dot Plot of the marker genes for each cluster. (C) Violin plot of the expression of all ISGs. (RNA counts added together).

Monocytes are a highly diverse immune cell type. scRNA-seq in combination with PolyI:C and LPS challenge allows us to dissect and interrogate the different functional roles of individual monocyte subtypes. We hypothesized that ISGs might be expressed on different subsets of monocytes. Therefore, we focused our analyses on the monocyte populations by reclustering them independently of other circulating immune cells to better understand their cellular diversity (Figures 3A and S2A). Reclustering resulted in 14 unique monocyte subclusters, each with its own characteristic markers (Figure 3B). The three original monocyte clusters (CD14 monocyte1, CD14 monocyte2, and CD16 monocyte) still separated upon reclustering, with each now having multiple smaller clusters (Figure S2B). While all subclusters expressed cytokines, chemokines, and ISGs after stimulation with PolyI:C (Figure S3A) or LPS (Figure S3B), one cluster (cluster 9) had particularly high ISG expression at baseline and in response to PolyI:C; expression of ISGs in this cluster remained low in overweight patients (Figure 3C). Conversely, most clusters robustly induced ISG expression in response to LPS, though cluster 9 was still the most responsive. Together, these data are consistent with the concept that monocytes are diversified in their individual responses to different stimuli, and all monocytes, even the highly responsive subcluster 9, were negatively affected in overweight patients.

### Pathway analyses reveal increased IFNG pathways in heavy drinkers

**Figure 4:**
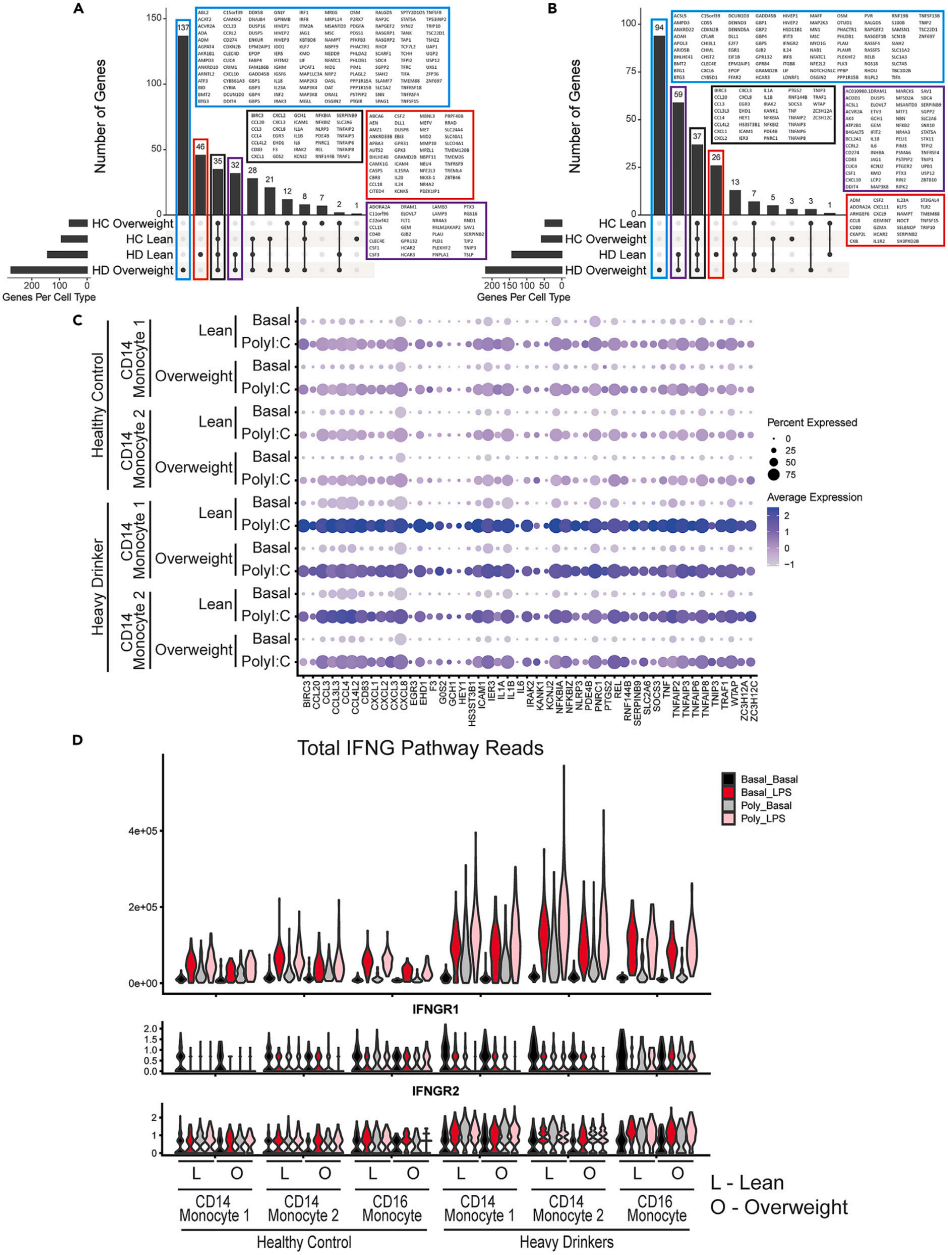
Heavy drinkers respond to PolyI:C with more robust pro-inflammatory and IFNG-associated genes (A and B) Upset plot summarizing differentially expressed genes upregulated in response to PolyI:C for CD14 monocyte 1 and 2, respectively. Black box—genes upregulated in all groups, blue box—only HD overweight, red box—only HC Lean, and purple box—HD lean and overweight. (C) Dot Plot showing relative expression and percentage of cells expressing gene that were commonly upregulated in response to PolyI:C. (D) Violin plot of the expression of all IFNG regulated genes (RNA counts added together), and the receptors for IFNG, IFNGR1, and IFNGR2.

To understand the general immune response, we measured differentially expressed genes for the major CD14 monocyte clusters (CD14 monocyte1 and CD14 monocyte2) in response to PolyI:C or LPS compared to basal control. For both CD14 monocyte clusters, many genes were commonly upregulated by PolyI:C or LPS (Figures 4A and 4B black boxes, Tables S5 and S6) regardless of heavy drinking or weight. Interestingly, in HD (lean and overweight), many more genes were upregulated by PolyI:C or LPS compared to HC (blue, red, and purple boxes). For example, in response to PolyI:C, 35 genes were upregulated in CD14 monocyte cluster 1 (Figure 4A), and 37 genes were upregulated in CD14 monocyte cluster 2 (Figure 4B) in all patient groups. While all of these genes were upregulated by PolyI:C in monocytes from HC and HD, lean and obese, their expression in HD was much higher than in HC, suggesting that these cells are more pro-inflammatory in HD (Figure 4C). The genes upregulated in response to PolyI:C or LPS include CC- and CXC-type chemokines and many NF-kB-associated genes and cytokines (black box). But in HD, in CD14 monocyte cluster 1,205 additional genes were upregulated: 137 in overweight HD (blue box), 46 in lean HD (red box), and 32 upregulated in lean and overweight HD (purple box). All of these genes were modestly, but not significantly, upregulated in HC monocytes (Figure S4). CD16 monocytes were not included in this analysis because there were not enough cells to analyze.

Pathway analyses of genes upregulated in response to PolyI:C consistently showed upregulation of many genes involved in innate immunity and inflammation in both CD14^+^ monocyte clusters (Figure S5). Focusing on anti-viral responses, pathway analysis revealed that monocytes from HD responded to PolyI:C with increased expression of more pro-inflammatory cytokine genes than HC (Figures 3 and S6). In particular, genes downstream of IFNG signaling (GO:0034341 list) were consistently upregulated in monocytes from HD, both lean and overweight (Figures 4D and S7). While IFNG itself was not detected in the single-cell data, IFNGR1 and IFNGR2 expression was higher in monocytes from HD (Figure 4D). Together, these results suggest that CD14^+^ monocytes from HD are more prone to pro-inflammatory gene expression and a bias toward type-II, IFNG signaling.

### Pretreatment with PolyI:C had little detectable effect on LPS responses

We also performed scRNA-seq on PBMCs treated with both PolyI:C and LPS, to better understand how prior challenges with PolyI:C would affect secondary LPS signaling. In HC, monocytes challenged with both PolyI:C and LPS were nearly indistinguishable from cells treated with LPS alone, having almost no differentially expressed genes for either lean or overweight patients (Table S7). Monocytes from HD did upregulate a few genes by the combination compared to LPS alone, including pro-inflammatory chemokines (CCL8 and CCL15) in the lean HD monocyte cluster 1, and cytokines (IL6 and IL20) in the overweight HD monocyte cluster 2. These data are consistent with the hypothesis that monocytes from HD have exacerbated pro-inflammatory responses, even in conditions that had little to no effect in healthy control cells.

## Discussion

During the COVID-19 pandemic, it has become clear that certain populations are at greater risk for severe disease, hospitalization, and death. The risk factors for adverse outcomes in COVID-19 patients included male gender, age, and body weight. Recent studies have also found that HD are also more prone to severe COVID-19.^6^^,^^7^ Both heavy drinking and obesity are associated with poor outcomes for a number of infectious diseases beyond COVID-19, including other viral and bacterial infections. In this study, we sought to understand how being overweight and heavy drinking can lead to perturbations in innate immune responses, specifically the balance of pro-inflammatory cytokines, chemokines, anti-viral genes, and type-I, -II, and -III interferons. scRNA sequencing in COVID-19 patients showed that the severity of disease directly correlates with transcriptomic profile;^31^ however, the effect of heavy alcohol drinking and overweight/obese status was not defined in these studies.

scRNA-seq allowed us to measure the gene expression responses to innate immune challenges, such as PolyI:C and LPS, in individual cells, and compare how these responses differ among HD and overweight patients. From these data, we made two separate conclusions about viral responses in monocytes: (1) being overweight leads to decreased expression of anti-viral genes, specifically ISGs that have specific roles in viral inhibition and (2) heavy drinking leads to increased expression of pro-inflammatory cytokines and chemokines and type-II IFNG-associated genes. Taken together, these results suggest that being overweight has negative effects on type-I and -III interferon signaling, leading to decreased anti-viral gene expression, while heavy alcohol consumption increases type-II interferons and inflammation. Thus, in HD who are also overweight, the balance between these different signaling pathways is shifted such that the immune system is unable to activate an anti-viral response but activates a heightened pro-inflammatory response. Together, they likely contribute to higher susceptibility and severity of disease, ultimately leading to worse outcomes.

Overweight/obesity increases susceptibility to a number of viral diseases in addition to COVID-19,^32^ including influenza,^33^ and other respiratory infections.^34^ From the proteomics data, we found that COVID-19 patients with obesity did not have many elevated signatures of inflammation, like pro-inflammatory cytokines and chemokines. Instead, our scRNA-seq data revealed reduced anti-viral gene expression in monocytes, such reductions would not have been detectable in proteomics data. Decreased anti-viral responses in patients with obesity have been characterized in some limited studies focused on PBMCs.^35^ Due to the COVID-19 pandemic, and the growing incidence of overweight and obesity worldwide, a greater interest has emerged to better understand how obesity alters the innate and adaptive immune systems.^4^^,^^36^ These studies find that obesity exacerbates inflammation associated with both increased adiposity and viral load.^37^^,^^38^ Another group found that obesity has a negative effect on many immune functions, but these responses differ based on age, with many of these defects much more pronounced among older COVID-19 patients.^39^ Finally, all of these studies are further compounded by the fact that many viral infections increase susceptibility to other bacterial and fungal infections, making the demands on the immune system even more complicated, and likely worsened by comorbidities such as obesity.^14^^,^^15^^,^^40^

Unfortunately, less is known about the role of alcohol consumption in COVID-19. HD have higher rates of severe COVID-19 and worse outcomes,^6^ and HD who are also obese had higher rates of mortality caused by COVID-19.^7^ While heavy drinking is associated with increased risk and severity of infection, especially viruses that target the liver,^41^ chronic alcohol consumption also leads to increased cytokine responses, resulting in increased inflammation despite the decreased ability to fight infections.

Less is known about the role of IFNG signaling among HD. In a murine model of alcohol dependence, IFNG signaling is increased,^42^ though the mechanism for this is unknown. In Rhesus macaque models of chronic heavy drinking, pro-inflammatory immune responses and increased cytokine production are heightened in PBMCs of heavy-drinking monkeys.^43^ Among the elevated cytokines in response to a bacterial challenge, IFNG secretion was higher in the heavy-drinking monkeys. While IFNG has some anti-viral functions, IFNG is associated with negative COVID-19 outcomes.^27^^,^^44^ Our data suggest that IFNG is elevated in HD in response to bacterial and viral challenges, suggesting that inhibitors of IFNG could be a novel therapeutic target to suppress inflammation in heavy drinking patients.

This study utilized scRNA-seq to understand how immune responses to PolyI:C and LPS differ between patients who are overweight and HD. Because of the financial constraints of these kinds of studies, these experiments were done on a small number of patients (n = 3 for all groups) and utilized only female subjects due to the known sex differences observed in terms of general immune responses during the COVID19 pandemic.^45^^,^^46^ Sex differences in the immune system will likely affect all of these results because immune cells from females have a much stronger interferon response to viral challenge than males. Moreover, females are more susceptible to the metabolic insults caused by being overweight and heavy drinking. Our results reflect how severe alcohol and weight can negatively impact innate immune cells and their responses; future studies are required to understand how these responses differ in males, who are more susceptible to viral infections.

### Limitations of the study

This study focused on understanding anti-viral and pro-inflammatory responses in immune cells from HD who were overweight. Our results help us to understand how these patients might be more susceptible to COVID-19 infection, but a more direct experiment with the actual COVID-19 virus might reveal more specific responses to the SARS-CoV2 virus that cannot be generalized. Additionally, this cohort of patients does not currently include a very large range for BMI among the HD, so we focused our study on the overweight HD (BMI between 25 and 30), while our overweight HC had a higher BMI (>30), making them clinically obese. Thus, it is likely much more dramatic results were observed in the obese HC, but it is interesting that many of these phenotypes were also observed in the overweight HD, suggesting the possibility that these immune changes affect overweight and obese patients to varying degrees. Future studies will need to look at how variations in patient populations, like BMI, alcohol consumption, and sex, affect these different immune factors.

### Conclusion

Using scRNA-seq, we find that increased body weight and heavy drinking have negative effects on viral response pathways in monocytes. People who are overweight had reduced anti-viral response genes activated by a viral mimic, while HD were much more pro-inflammatory. During the COVID-19 pandemic, people who were overweight, HD, or both were far more likely to experience severe disease and negative outcomes. Our data suggest that the ability to fight viral infections is perturbed among the overweight and HD via different immunological mechanisms. These data suggest that different treatment strategies might be necessary for these patients, and that getting both an assessment of their weight and a history of alcohol use is critical to understanding their risk for severe disease.

## STAR★Methods

### Key resources table

**Table:** 

REAGENT or RESOURCE	SOURCE	IDENTIFIER
**Antibodies**		

Totalseq A antibodies	Biolegend	https://www.biolegend.com/en-us/protocols/totalseq-a-antibodies-and-cell-hashing-with-10x-single-cell-3-reagent-kit-v3-3-1-protocol

**Biological samples**		

Cryopreserved PBMCs isolated from heavy drinkers and healthy controls	Cleveland Clinic and MetroHealth Hospital	N/A

**Chemicals, peptides, and recombinant proteins**		

PolyI:C	Sigma-Aldrich	P1530, Lot:089M4092V
LPS	Sigma-Aldrich	Escherichia coli strain L2654(O26:B6) Lot 0000089955
Cell Staining Buffer	Biolegend	420201
Human TruStain FcX Blocking Reagent	Biolegend	N/A

**Critical commercial assays**		

10x Chromium v3.1	10x	PN-1000121

**Deposited data**		

Plasma Proteomics from COVID-19 infected and non-infected patients	MGH Emergency Dept COVID-19 cohort with Olink Proteomics	https://doi.org/10.1016/j.xcrm.2021.100287
scRNA-seq of PBMCs from heavy drinkers and health controls challenged with PolyI:C and LPS	This Study	NCBI SRA - PRJNA891850
Code used to analyze data and generate figures	This Study	https://github.com/atomadam2/PolyIC_HeavyDrinkers

**Oligonucleotides**		

SI-PCR primer AATGATACGGCGACCACCGAGATCTACACTCTTT CCCTACACGACGC∗T∗C	Biolegend totalseq Protocol	https://www.biolegend.com/en-us/protocols/totalseq-a-antibodies-and-cell-hashing-with-10x-single-cell-3-reagent-kit-v3-3-1-protocol
HTO additive primer v2 GTGACTGGAGTTCAGACGTGTGCTCTTCCGAT∗C∗T	Biolegend totalseq Protocol	https://www.biolegend.com/en-us/protocols/totalseq-a-antibodies-and-cell-hashing-with-10x-single-cell-3-reagent-kit-v3-3-1-protocol
D701_LONG CAAGCAGAAGACGGCATACGAGATCGAGTAATGT GACTGGAGTTCAGACGTGTGCTCTTCCGAT∗C∗T	Biolegend totalseq Protocol	https://www.biolegend.com/en-us/protocols/totalseq-a-antibodies-and-cell-hashing-with-10x-single-cell-3-reagent-kit-v3-3-1-protocol
D702_LONG CAAGCAGAAGACGGCATACGAGATTCTCCGGAGT GACTGGAGTTCAGACGTGTGCTCTTCCGAT∗C∗T	Biolegend totalseq Protocol	https://www.biolegend.com/en-us/protocols/totalseq-a-antibodies-and-cell-hashing-with-10x-single-cell-3-reagent-kit-v3-3-1-protocol
D703_LONG CAAGCAGAAGACGGCATACGAGATAATGAGCGGT GACTGGAGTTCAGACGTGTGCTCTTCCGAT∗C∗T	Biolegend totalseq Protocol	https://www.biolegend.com/en-us/protocols/totalseq-a-antibodies-and-cell-hashing-with-10x-single-cell-3-reagent-kit-v3-3-1-protocol
D704_LONG CAAGCAGAAGACGGCATACGAGATGGAATCTCGT GACTGGAGTTCAGACGTGTGCTCTTCCGAT∗C∗T	Biolegend totalseq Protocol	https://www.biolegend.com/en-us/protocols/totalseq-a-antibodies-and-cell-hashing-with-10x-single-cell-3-reagent-kit-v3-3-1-protocol
D705_LONG CAAGCAGAAGACGGCATACGAGATTTCTGAATGT GACTGGAGTTCAGACGTGTGCTCTTCCGAT∗C∗T	Biolegend totalseq Protocol	https://www.biolegend.com/en-us/protocols/totalseq-a-antibodies-and-cell-hashing-with-10x-single-cell-3-reagent-kit-v3-3-1-protocol
D706_LONG CAAGCAGAAGACGGCATACGAGATACGAATTCGT GACTGGAGTTCAGACGTGTGCTCTTCCGAT∗C∗T	Biolegend totalseq Protocol	https://www.biolegend.com/en-us/protocols/totalseq-a-antibodies-and-cell-hashing-with-10x-single-cell-3-reagent-kit-v3-3-1-protocol
D707_LONG CAAGCAGAAGACGGCATACGAGATAGCTTCAGGT GACTGGAGTTCAGACGTGTGCTCTTCCGAT∗C∗T	Biolegend totalseq Protocol	https://www.biolegend.com/en-us/protocols/totalseq-a-antibodies-and-cell-hashing-with-10x-single-cell-3-reagent-kit-v3-3-1-protocol
D708_LONG CAAGCAGAAGACGGCATACGAGATGCGCATTAGT GACTGGAGTTCAGACGTGTGCTCTTCCGAT∗C∗T	Biolegend totalseq Protocol	https://www.biolegend.com/en-us/protocols/totalseq-a-antibodies-and-cell-hashing-with-10x-single-cell-3-reagent-kit-v3-3-1-protocol

**Software and algorithms**		

Cellranger (v3.0.2)	10x	https://support.10xgenomics.com/single-cell-gene-expression/software/overview/welcome
Seurat (3.1.1)	Stuart, Hafemeister, Satija	https://doi.org/10.1016/j.cell.2019.05.031 https://doi.org/10.1186/s13059-019-1874-1

**Other**		

FlowMi Cell Strainers	Bel-Art	H13680-0040

### Resource availability

#### Lead contact

Further information and requests for resources and reagents should be directed to and will be fulfilled by the lead contact, Adam Kim, adkim@uchc.edu.

#### Materials availability

This study did not generate new unique reagents.

### Experimental model and study participant details

#### Alcohol-related hepatitis and healthy control patient selection

HC or HD with an AUDIT score greater than >16 were recruited from the Clinical Research Unit at the Cleveland Clinic or MetroHealth Hospital based on medical history and physical examination. All patient samples were collected and cryopreserved prior to the COVID-19 pandemic. Detailed clinical information on the patients used for single cell analysis are presented in Table S2.

#### Study approval

The study protocol was approved by the institutional review board for the Protection of Human Subjects in Research at the Cleveland Clinic and MetroHealth Hospital, Cleveland. All methods were performed in accordance with the internal review board’s guidelines and regulations, and written, informed consent was obtained from all subjects.

### Method details

#### Plasma proteomics from lean and overweight patients with or without COVID-19 infection

Plasma proteomics from COVID-19 infected and non-infected patients were analyzed using publicly available data from^28^ [Data provided by the MGH Emergency Dept COVID-19 Cohort with Olink Proteomics]. Briefly, protein expression related to antiviral response pathways were analyzed in COVID-19 and non-infected subjects and stratified/classified based on BMI as lean (BMI <25), overweight (BMI between 25 and 30), and obese (BMI >30). Acquired data from hospital admission (Day 0) based on experimental design was use for this analysis. Patients with immunocompromised response were removed from the analysis. Circulating protein levels were compared between the 4 groups using One Way ANOVA statistical test and plotted in violin plots for data visualization.

#### Isolation of human peripheral blood mononuclear cells

PBMCs were isolated from human blood as previously described.^47^^,^^48^ Isolation of mononuclear cells was performed by density gradient centrifugation on Ficoll-Paque PLUS (GE Healthcare, Uppsala, Sweden). 1 mL of freshly collected Buffy Coat was mixed at a ratio of 1:5 (v/v) with phosphate buffered saline (PBS) at 37°C and divided and layered onto 8 mL Ficoll-Paque PLUS in two 15 mL conical centrifuge tubes. After centrifugation at 400 × g for 30 min at 20°C (no brake), buffy coat fractions were collected, pooled, resuspended in culture media (Roswell Park Memorial Institute (RPMI)-1640 supplemented with 100 μM Penicillin-streptomycin and 10% fetal bovine serum (FBS)), and centrifuged at 400 × g for 15 min at 20°C. The pellets were resuspended in 8 mL of culture media, counted, and again centrifuged at 400 × g for 8 min at 20°C. Cells were then cryo-preserved by resuspending in freezing media (50% culture media, 40% FBS, 10% dimethyl sulfoxide (DMSO)) at a concentration of 1.5 × 10^6^ cells/mL, and allowed to freeze slowly to −80°C in a styrofoam container. For long-term preservation, cells were stored in liquid nitrogen.

#### Challenging peripheral blood mononuclear cells with PolyI:C and lipopolysaccharide *ex vivo*

For single-cell experiments, cryopreserved PBMCs were selected from female patients of the following groups: three lean and three overweight HC and three lean and three overweight HD. Cryopreserved PBMCs were thawed at 37°C, then added slowly to 8 mL of warm culture media, as previously described.^48^ After centrifugation at 400 × g for 8 min at 20°C, cells were resuspended and cultured in 96-well plates at a cell density of ∼50,000 cells/well in a humidified atmosphere (5% CO2, 37°C). After 18 h, cells were washed with media and challenged with or without 1.5 μg/mL PolyI:C (P1530, Lot:089M4092V, Sigma-Aldrich) for 2 h, or with or without 10 ng/mL LPS (Escherichia coli strain L2654(O26:B6) Lot 0000089955, Sigma-Aldrich) for 1 h. To resuspend, cells were washed one time with PBS, then incubated on ice with PBS +2.5 mM ethylene diamine tetraacetic acid (EDTA) for 30 min. After gently resuspending cells by pipetting, cells were spun in Eppendorf tubes at 300× g for 5 min. Cells were resuspended in Cell Staining Buffer (Biolegend, San Diego, CA) prior to multiplexing.

#### Multiplexing of peripheral blood mononuclear cells prior to Single-cell RNA-sequencing

Multiplexing was performed following Biolegend’s v3-3-1 cell hashing protocol using Biolegend Totalseq A antibodies. PBMCs in cell stain buffer were blocked with Human TruStain FcX Blocking Reagent (Biolegend) for 10 min at 4°C. Then we added a unique TotalSeq A antibody for each reaction (Biolegend) and incubated for 30 min at 4°C. Cells were washed twice with cell stain buffer and spun at 830 x g at 4°C for 4 min. Samples from the same patient were then pooled together, and filtered through FlowMi Cell Strainers (Bel-Art, South Wayne, NJ). Cells were washed with PBS +0.04% bovine serum albumin, resuspended, and counted for 10x sequencing. Viability was >95% for all samples. Gel beads in emulsion and libraries were performed according to manufacturer’s instructions (Chromium v3.1). Libraries were quantified using an Agilent Bioanalyzer (Agilent Technologies, Santa Clara, CA), then pooled. Multiplex Hashing libraries were generated according to the original protocol,^49^ and pooled and sequenced alongside RNA-seq libraries using a NovaSeq6000.

### Quantification and statistical analysis

#### Clustering and differential expression

Sequencing data were aligned to the Human genome (GRC38, release 93) using cellranger (v3.0.2). All gene expression and clustering analyses were performed using Seurat (3.1.1) as previously described.^48^^,^^50^ Briefly, all samples were first normalized using SCTransform and then filtered to remove low quality cells (nFeature_RNA<4000, nFeature_RNA>200, percent.mt < 20, which removes doublets, cells with low reads, and cells with high mitochondrial content)).^51^ All samples were combined using the PrepSCTIntegration and FindIntegrationAnchors functions to find common anchor genes in all samples for all cell types, and then integrated using the IntegrateData function, with all normalizations using the SCT transformed data.^50^^,^^52^ Clustering was performed using RunPCA and RunUMAP, and clusters were identified using FindNeighbors and FindClusters. Differentially expressed genes were measured using Libra^53^ in R, which utilized pseudobulk analyses of individual sample clusters, then EdgeR was used for differential expression calculations. Differential expression calculations were not performed on CD16_monocyte, CD8_T cell8, and DC_cell clusters because there were not enough cells (less than 1200 total cells in all samples). A statistical cutoff of p adj<0.05 was used for all analyses. All pathway analyses were performed using Metascape from lists of significant differentially expressed genes.^54^

#### Ethical approval and consent to participate

The study protocol was approved by the Institutional Review Board for the Protection of Human Subjects in Research at the Cleveland Clinic and MetroHealth Hospitals, Cleveland. All methods were performed in accordance with the IRB’s guidelines and regulations and written informed consent was obtained from all subjects.

## Data Availability

• The scRNA-seq data for this study can be found at National Center for Biotechnology Information Gene Expression Omnibus under accession number [PRJNA891850]. • All scripts used for analyses, differential expression results, for all cell types, and figure generation can be found at the author’s github (https://github.com/atomadam2/PolyIC_HeavyDrinkers). • Any additional information required to reanalyze the data reported in this paper is available from the lead contact upon request.

## Supplemental information

Document S1. Figures S1–S7, Tables S1, S2, S4, and S7

Table S3. Number of cells for each cluster within each patient treatment sample, related to Figure 2

Table S5. Differentially expressed genes in response to PolyI:C, related to Figure 4

Table S6. Differentially expressed genes in response to LPS, related to Figure 4
