# Supplementary material for: Altered anti-viral immune responses in monocytes in overweight heavy drinkers
Source: iScience. 2023 Jun 15;26(7):107133. doi: 10.1016/j.isci.2023.107133 (PMC10268809; doi:10.1016/j.isci.2023.107133)
Supplement: Document S1. Figures S1–S7, Tables S1, S2, S4, and S7 [file mmc1.pdf]

iScience, Volume *26*

Supplemental information

Altered anti-viral immune responses

in monocytes in overweight heavy drinkers

Adam Kim, Martí Ortega-Ribera, Megan R. McMullen, Annette Bellar, Moyinoluwa Taiwo, Vai Pathak, David Streem, Jaividhya Dasarathy, Nicole Welch, Srinivasan Dasarathy, Vidula Vachharajani, and Laura E. Nagy

Supplemental Figure 1

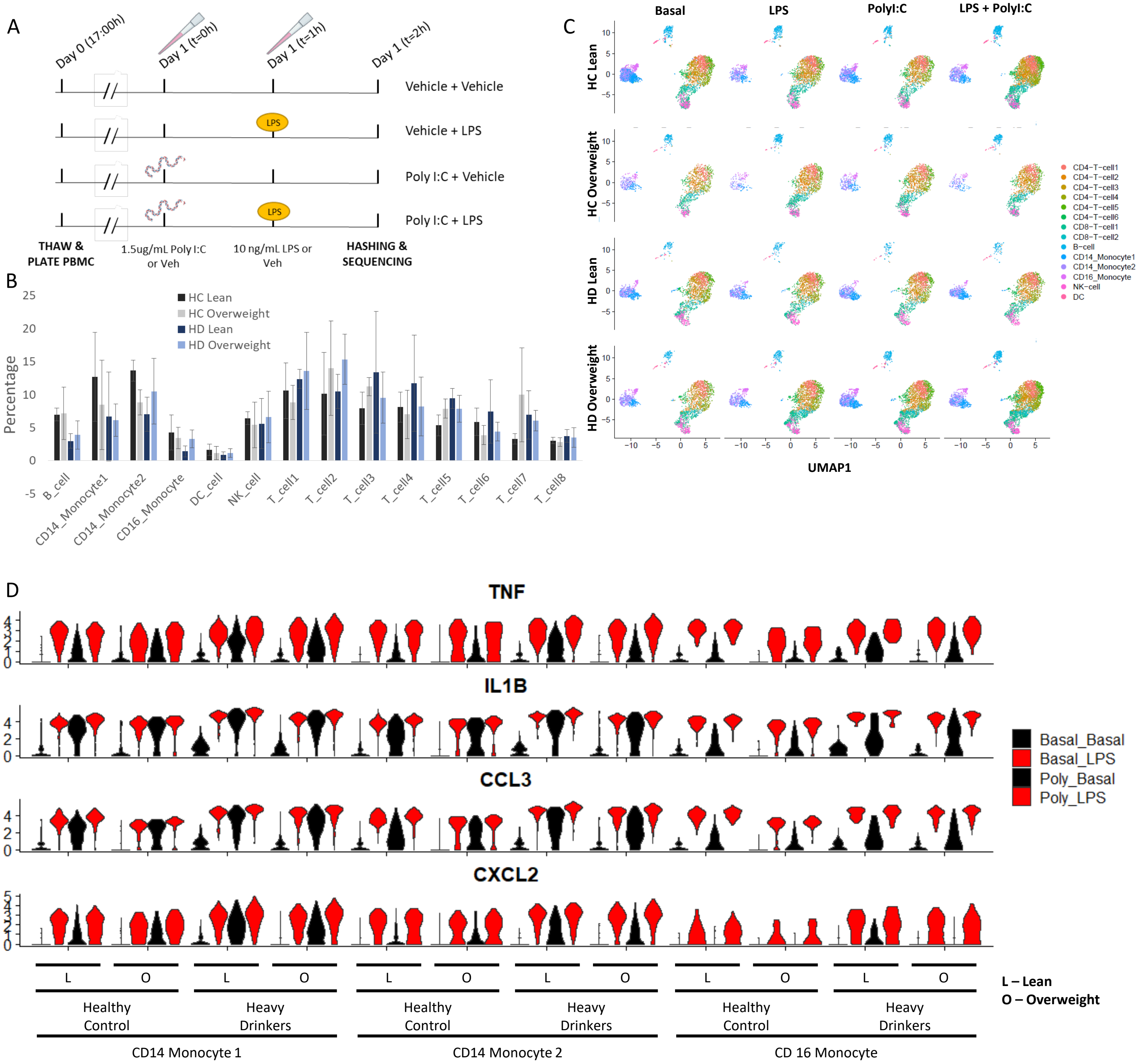

Supplemental Figure 1: Single-cell RNA-seq of PBMCs challenged with PolyI:C and LPS, related to Figure 2 and STAR Methods – A) Schematic showing experimental design. PBMCs isolated from lean and overweight HD and HC were challenged *ex vivo* with and without PolyI:C for two hours, LPS for 1 hour or their combination prior to scRNA-seq. Vehicle (PBS) treatment was used as control. B) Bar chart summarizing the percentage of each cell type/cluster from the scRNA-seq data. Vales are the mean and error bars are the standard deviation. C) UMAP plots for the entire scRNA-seq dataset split by disease and treatment. D) Violin plots showing expression of pro-inflammatory cytokines (TNF, IL1B) and chemokines (CCL3 and CXCL2) in response to PolyI:C and LPS.

Supplemental Figure 2

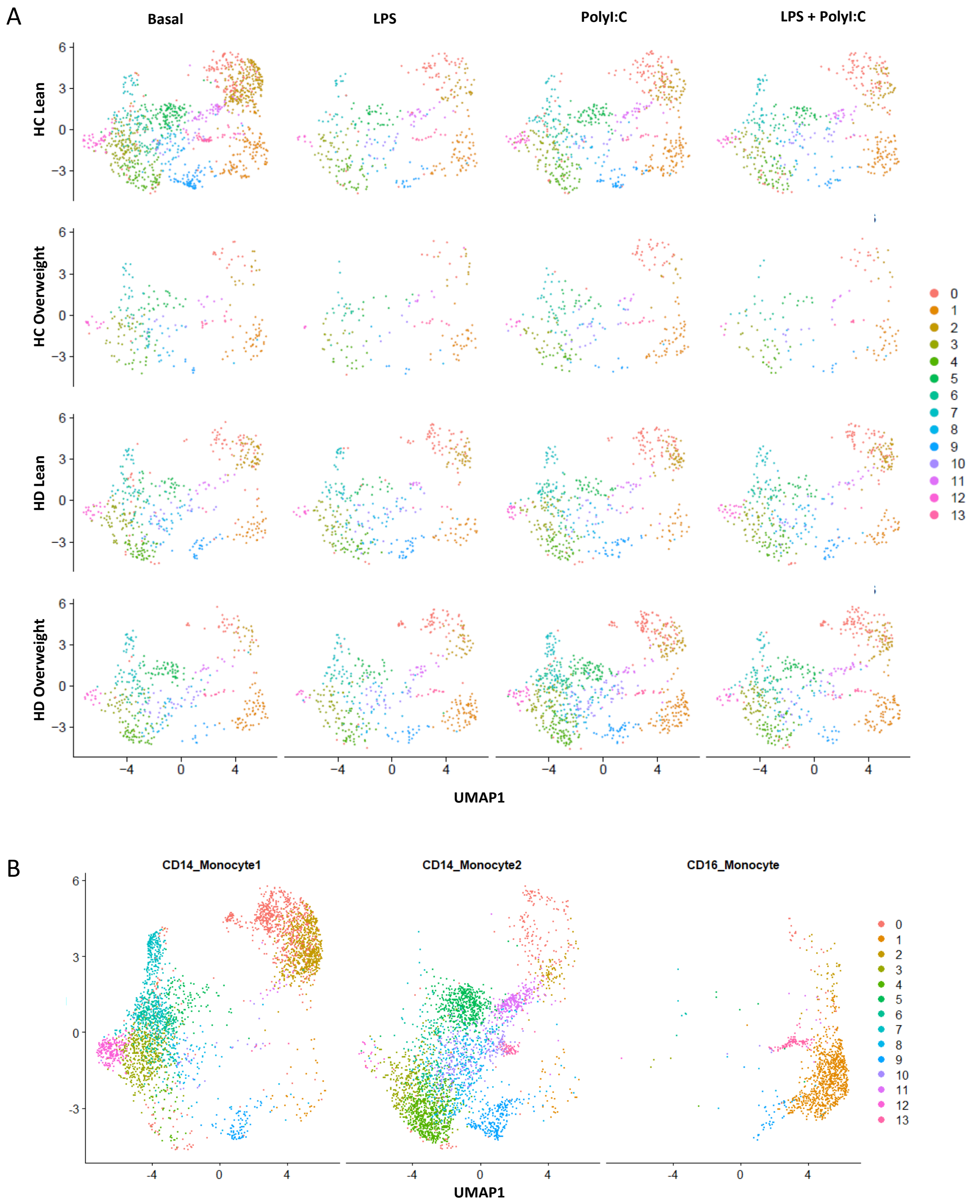

Supplemental Figure 2: Clustering of the monocyte only subsets, related to Figure 3 – A) UMAP plots for the refined monocyte subclustering analysis split by disease and treatment. B) UMAP plots for the refined monocyte subclustering analysis split by the original monocyte subclusters

Supplemental Figure 3

A

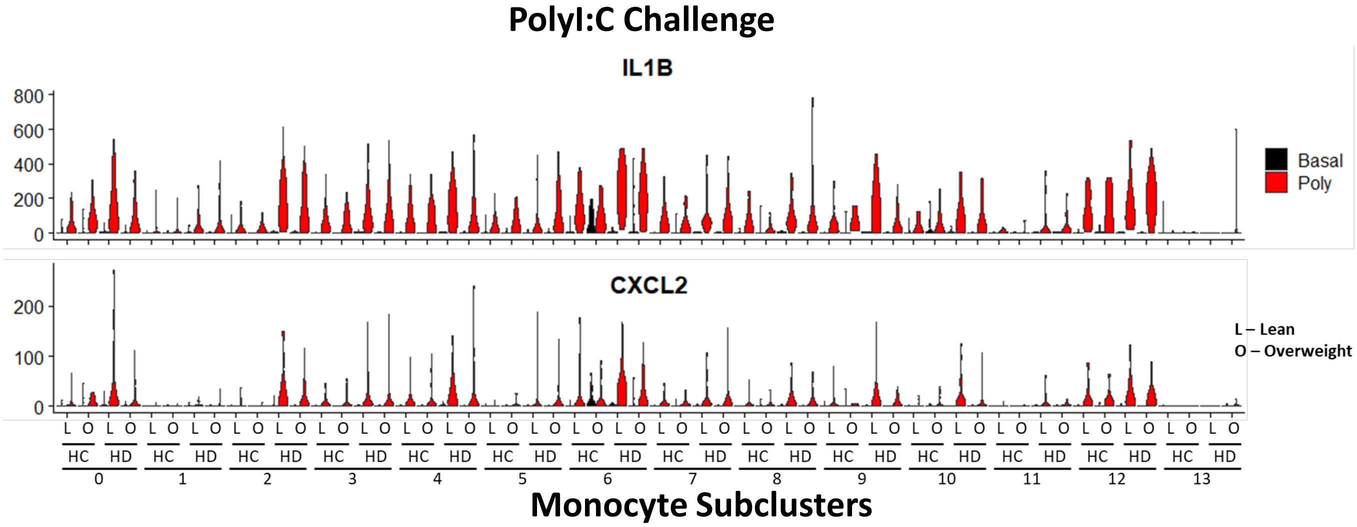

B

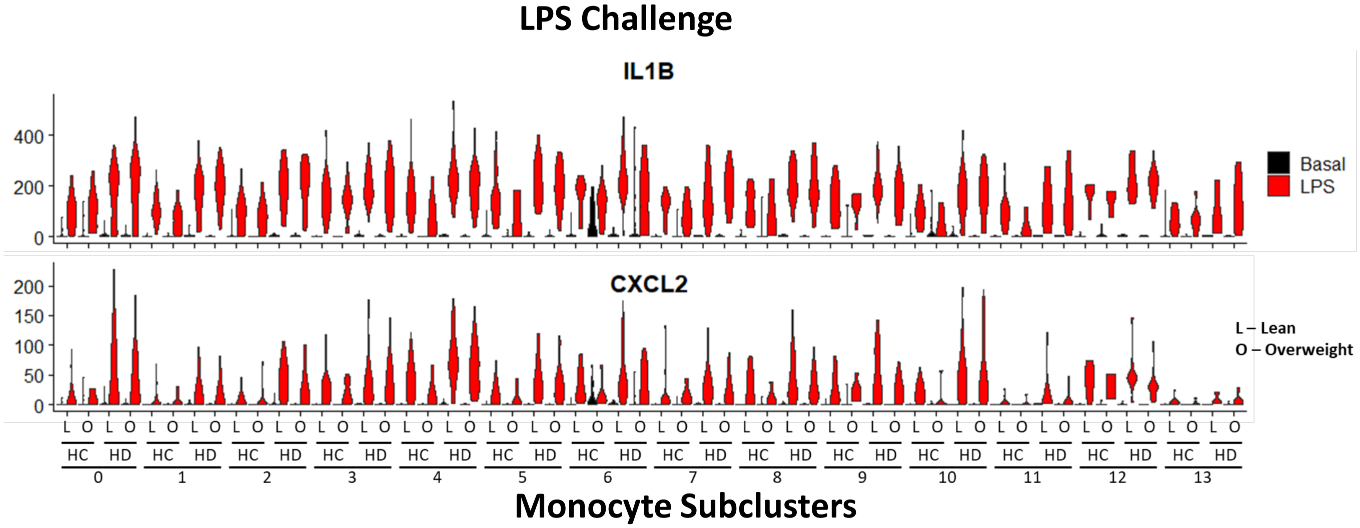

Supplemental Figure 3: Expression key cytokines and chemokines after PolyI:C and LPS, related to Figure 3 – Violin plots showing expression within the more refined monocyte subclusters of the pro-inflammatory cytokine IL1B and chemokine CXCL2 in response to A) PolyI:C and B) LPS.

Supplemental Figure 4

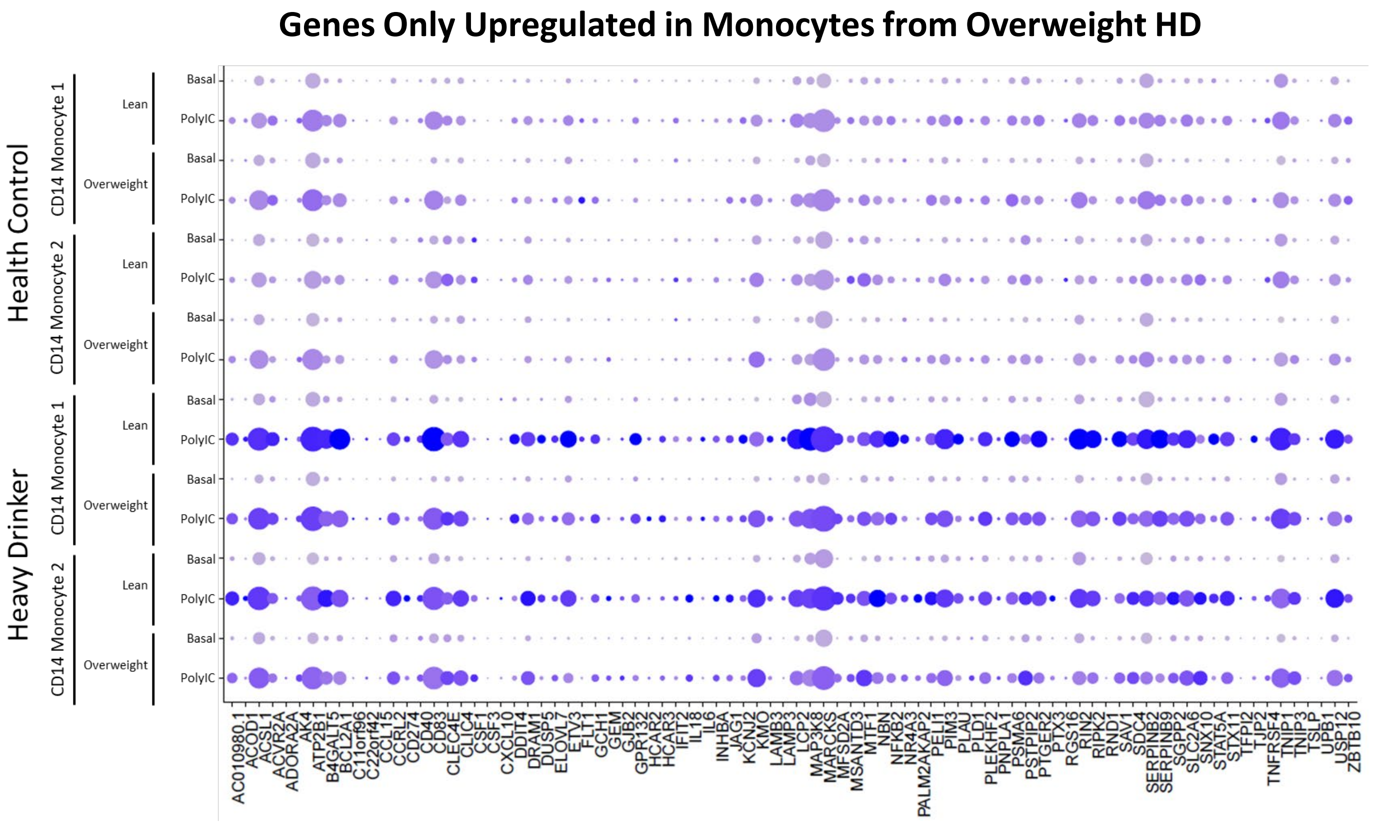

Supplemental Figure 4: Expression of genes upregulated in HD monocytes, related to Figure 4 – A) Dot Plot showing relative expression and percentage of cells expressing genes that were only upregulated in monocytes from heavy drinkers in response to PolyI:C.

Supplemental Figure 5

A **CD14 Monocyte Cluster 1**

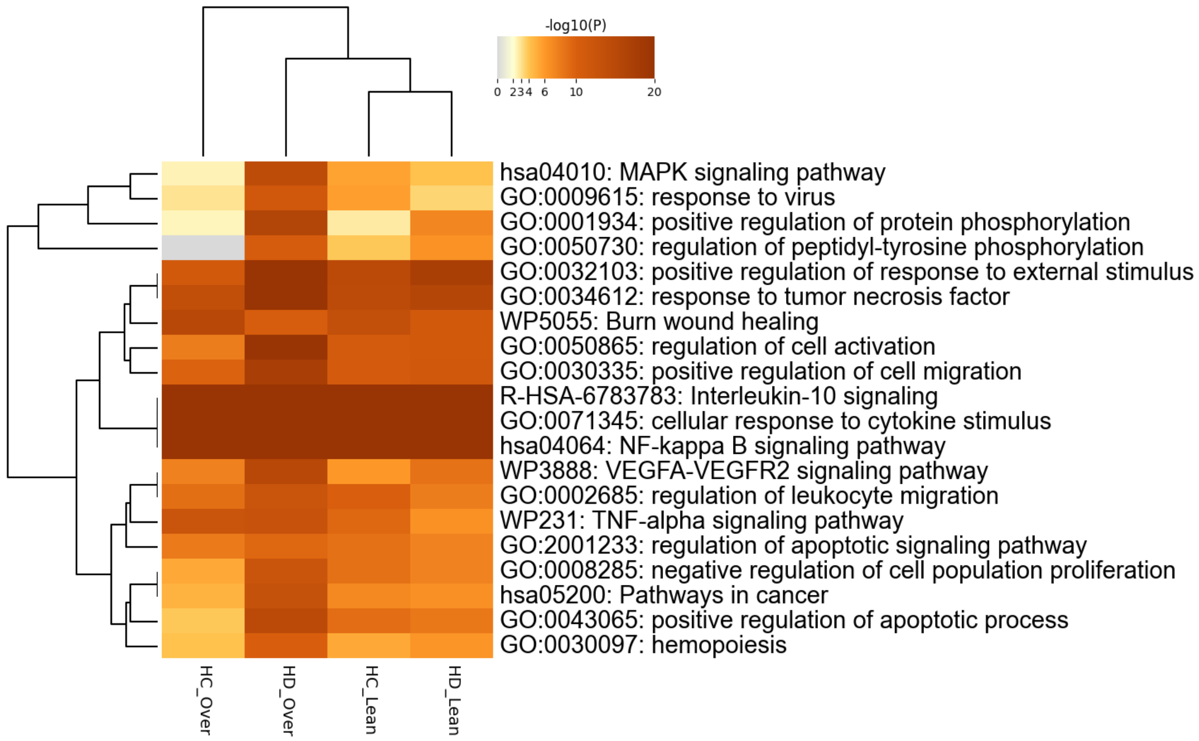

B **CD14 Monocyte Cluster 2**

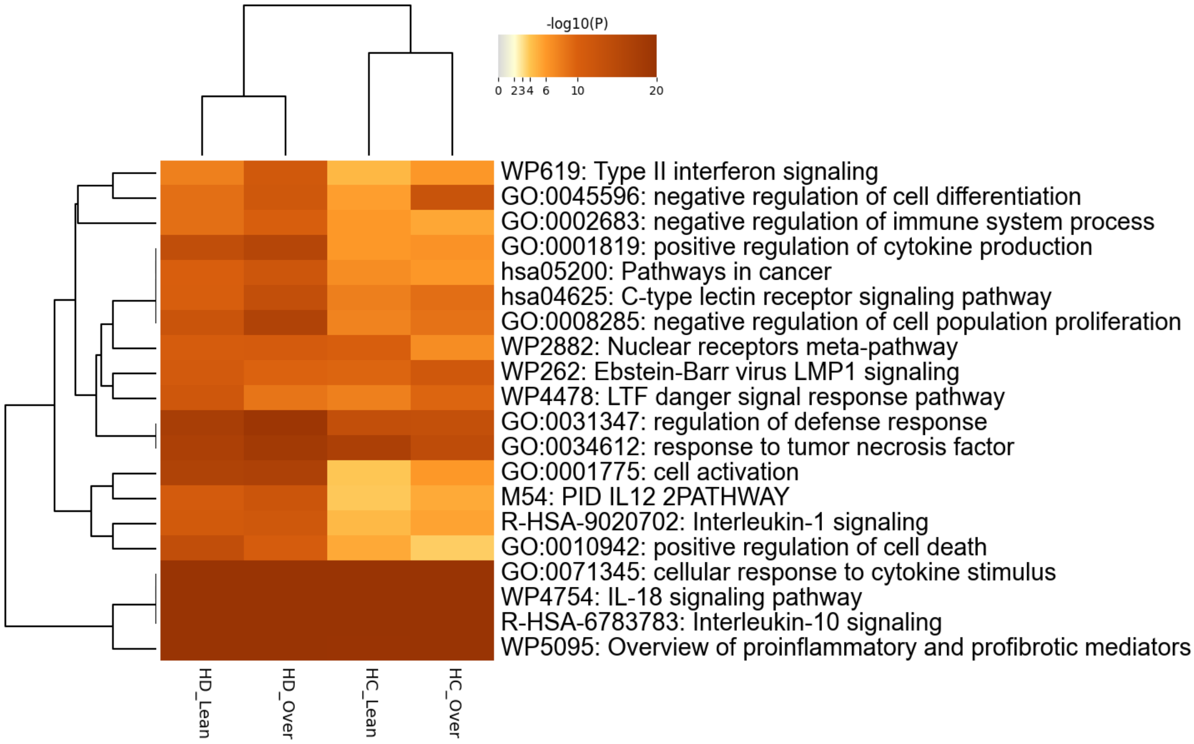

Supplemental Figure 5: Pathways upregulated in Monocytes after PolyI:C, related to Figure 4 – Pathway Analysis of genes upregulated after challenge with PolyI:C in A) CD14 Monocyte Cluster 1 and B) CD14 Monocyte Cluster 2

Supplemental Figure 6

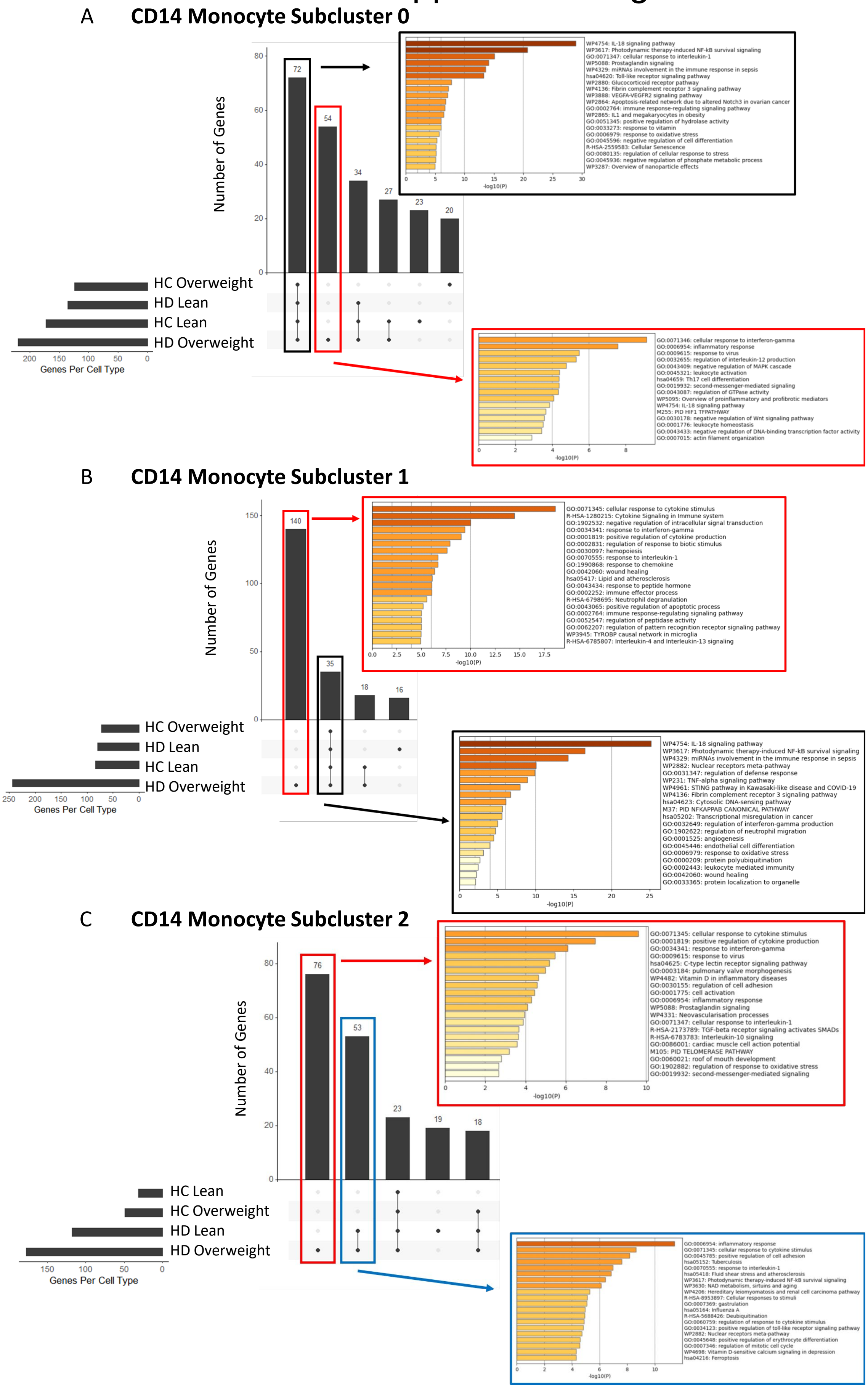

Supplemental Figure 6: Pathways upregulated in Monocytes after PolyI:C, related to Figure 4 – Pathway Analysis of genes upregulated after challenge with PolyI:C in the more refined Monocyte subclusters A) CD14 Monocyte Subcluster 1, B) CD14 Monocyte Subcluster 2 and C) CD14 Monocyte Subcluster 3

Supplemental Figure 7

IFNG Signaling Genes

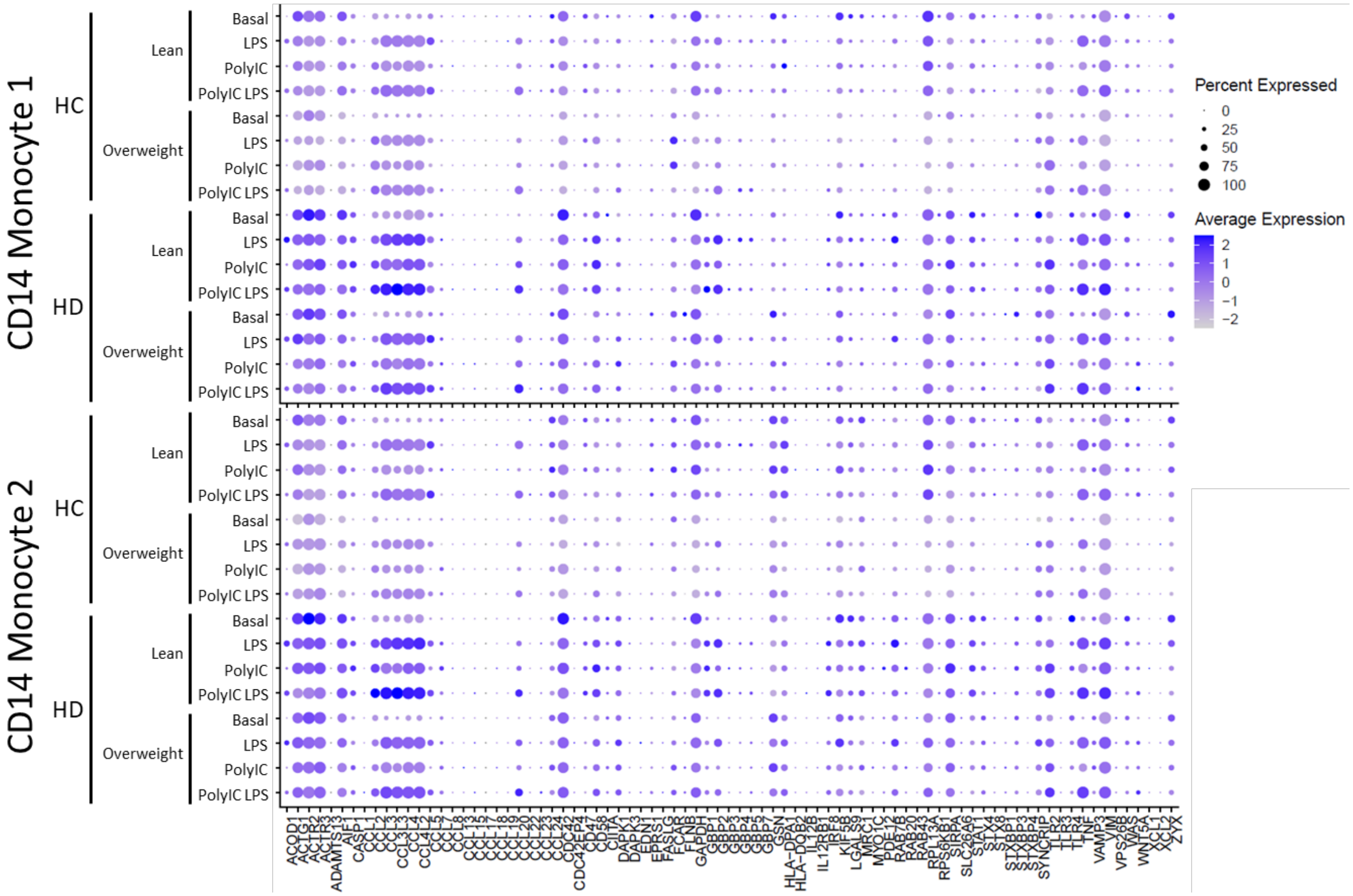

Supplemental Figure 7: Expression of genes related to IFNG signaling, related to Figure 4 – Dot Plot showing relative expression and percentage of cells expressing IFNG pathway genes (GO:0034341) in monocytes from heavy drinkers in response to PolyI:C and/or LPS.

Table 1: Summary of Clinical Data, related to STAR Methods				
Mean (Range)	**Healthy Control Lean, n=3**	**Heavy Drinker Lean, n=3**	**Healthy Control Overweight, n=3**	**Heavy Drinker Overweight, n=3**
Age (years)	47 (27-57)	40 (27-61)	57 (52-62)	41 (28-58)
BMI	22 (18-24)	22 (20-24)	37 (30-45)	29 (27-30)
AST (U/L)	22 (14-27)	29 (18-41)	16 (12-20)	29 (21-30)
ALT (U/L)	23 (13-35)	26 (16-45)	18 (15-20)	37 (21-37)
Serum Albumin (g/dL)	4.4 (4.4-4.4)	4.3 (4.1-4.5)	4.0 (3.9-4.0)	4.1 (3.5-4.5)
AUDIT Score	(-)	21 (16-30)	(-)	25 (20-27)

Supplemental Table 2: Clinical data for patient samples used for single-cell RNA-seq, related to STAR Methods																	
**Patient**	**Disease**	**Weight Group**	**Day of Seq**	**Age**	**Gender**	**Race**	**BMI**	**Weight Group**	**Bilirubin**	**AST**	**ALT**	**Creatanine**	**Albumin**	**ALP**	**Protein**	**Sodium**	**AUDIT**
P50_42	HC	Lean	Day3	57	Female	White	22.22	Lean	0.2	25	35	0.75	4.4				
P50_94	HC	Lean	Day2	57	Female	White	18.06	Lean	0.5	27	22	0.85	4.4	52	6.2	143	
P50_102	HC	Lean	Day4	27	Female	White	24.3	Lean	0.2	14	13	0.88	4.4	42	7	144	
MH_39	HD	Lean	Day2	27	Female	White	21.4	Lean		18	16	0.51	4.3	59	6.9	140	18
MH_42	HD	Lean	Day3	31	Female	Black	24.34	Lean		29	16		4.1	86	6.9		16
MH_58	HD	Lean	Day5	61	Female	Black	19.72	Lean		41	45	0.8	4.5	78	6.6	138	30
P50_36	HC	Overweight	Day1	62	Female		35.45	Overweight									
P50_49	HC	Overweight	Day3	52	Female	White	45.14	Overweight	0.4	12	15	0.81	3.9				
P50_78	HC	Overweight	Day2	58	Female	White	30.1	Overweight	0.4	20	20	0.69	4			142	
P50_133	HD	Overweight	Day1	28	Female	White	29.95	Overweight	0.8	30	28	0.81	4.5	34	7.4	138	20
MH_37	HD	Overweight	Day2	36	Female	White	27.46	Overweight		37	59		3.5	66	7		27
MH_64	HD	Overweight	Day5	58	Female	Black	28.79	Overweight		21	25		4.4	45	7.6		27

Supplemental Table 4: Differentially Expressed Genes in CD14 Monocyte Clusters at Baseline, related to Figure 2

CD14 Monocyte 1 - HC vs HD			
gene	avg_logFC	p_val	p_val_adj
TREML4	-4.7156	4.69E-05	0.032084
F13A1	-3.89643	2.60E-07	0.000675
SELENOP	-3.82796	7.11E-05	0.044054
CXCL10	-3.74465	1.63E-08	7.09E-05
PF4	-3.62391	8.43E-11	1.10E-06
IGHA2	-3.37697	1.44E-06	0.002085
PF4V1	-3.0751	3.71E-06	0.00402
TUBB1	-3.07397	3.11E-07	0.000675
PRKAR2B	-2.97195	1.01E-05	0.009411
CAVIN2	-2.62807	2.48E-06	0.003227
NRGN	-2.61399	3.12E-07	0.000675
RGS18	-2.44095	1.08E-06	0.001758
GNG11	-1.69166	6.73E-05	0.043767
SERPINB2	1.701868	3.86E-05	0.027893
FGL2	1.807482	3.65E-05	0.027893
ISG15	2.439704	3.57E-06	0.00402
CES1	2.464948	5.42E-06	0.005425
IFI44L	3.212435	1.92E-05	0.016622
PTGS2	3.585519	1.34E-08	7.09E-05
MARCO	4.036809	3.09E-05	0.025126
SCG5	5.464955	4.80E-07	0.000892

CD14 Monocyte 2 - HC vs HD			
gene	avg_logFC	p_val	p_val_adj
TREML4	-4.92651	9.41E-06	0.011679
AOAH	2.329951	3.92E-06	0.006231
MT1H	2.793044	1.01E-05	0.011679
SPP1	3.117279	4.67E-05	0.04574
CSF1	3.190889	2.73E-07	0.000867
APOBEC3A	3.399914	2.62E-06	0.005546
TNFSF10	3.55659	5.27E-05	0.0467
INHBA	3.634609	5.81E-08	0.000369
IFIT2	3.638712	1.47E-07	0.000622
CCL22	3.70441	7.71E-06	0.010902
IFIT1	4.057774	4.95E-07	0.00126
TNFSF15	5.13162	5.51E-05	0.0467
IFI27	5.235036	2.83E-05	0.029999
SCG5	6.66624	7.25E-10	9.22E-06
CXCL11	7.043336	3.33E-06	0.006051

Neg LogFC is associated with higher expression in Heavy

Supplemental Table 7: Differentially Expressed Genes between LPS vs PolyI:C/LPS, related to Figure 4

CD14 Monocyte 1 - HC Lean

gene	avg_logFC	p_val	p_val_adj
TNFSF15	-4.09	0.00	0.01
IGHA1	-3.34	0.00	0.02

CD14 Monocyte 1 - HC Overweight

gene	avg_logFC	p_val	p_val_adj

CD14 Monocyte 1 - HD Lean

gene	avg_logFC	p_val	p_val_adj
CCL8	-5.90	0.00	0.00
CCL15	-5.78	0.00	0.00
DKK2	-5.65	0.00	0.00
TSLP	-5.27	0.00	0.04
FAM102B	2.20	0.00	0.04
PFKFB4	5.13	0.00	0.04

CD14 Monocyte 1 - HD Overweight

gene	avg_logFC	p_val	p_val_adj
TFPI2	-7.94	0.00	0.00
IGHM	-6.12	0.00	0.00
CSF2	-4.24	0.00	0.00
PALM2AKAP2	-3.65	0.00	0.00
DPYSL3	-2.97	0.00	0.04
TMIGD3	5.21	0.00	0.04

CD14 Monocyte 2 - HC Lean

gene	avg_logFC	p_val	p_val_adj

CD14 Monocyte 2 - HC Overweight

gene	avg_logFC	p_val	p_val_adj
FABP4	4.31	0.00	0.01

CD14 Monocyte 2 - HD Lean

gene	avg_logFC	p_val	p_val_adj
TSLP	-5.50	0.00	0.04
TFPI2	-3.15	0.00	0.04

CD14 Monocyte 2 - HD Overweight

gene	avg_logFC	p_val	p_val_adj
CCL1	-6.64	0.00	0.00
IL20	-4.37	0.00	0.00
MMP7	-3.59	0.00	0.00
IL6	-3.11	0.00	0.00
TFPI2	-3.07	0.00	0.00
PTX3	-3.04	0.00	0.00
CSF2	-2.46	0.00	0.00
PPBP	-2.12	0.00	0.01
ANKRD1	2.54	0.00	0.00
LPAR6	3.34	0.00	0.01
CCDC170	3.92	0.00	0.03
SPIB	6.44	0.00	0.00
